# Comparative transcriptomic profile of tolerogenic dendritic cells differentiated with vitamin D3, dexamethasone and rapamycin

**DOI:** 10.1038/s41598-018-33248-7

**Published:** 2018-10-08

**Authors:** Juan Navarro-Barriuso, María José Mansilla, Mar Naranjo-Gómez, Alex Sánchez-Pla, Bibiana Quirant-Sánchez, Aina Teniente-Serra, Cristina Ramo-Tello, Eva M. Martínez-Cáceres

**Affiliations:** 1 Germans Trias i Pujol University Hospital and Research Institute, Immunology Division, Badalona, 08916 Spain; 2 Universitat Autònoma de Barcelona, Department of Cellular Biology, Physiology and Immunology, Cerdanyola del Vallès, 08193 Spain; 3 University of Barcelona, Department of Statistics, Barcelona, 08028 Spain; 4 Germans Trias i Pujol University Hospital, Department of Neurosciences, Multiple Sclerosis Unit, Badalona, 08916 Spain

## Abstract

Tolerogenic dendritic cell (tolDC)-based therapies have become a promising approach for the treatment of autoimmune diseases by their potential ability to restore immune tolerance in an antigen-specific manner. However, the broad variety of protocols used to generate tolDC *in vitro* and their functional and phenotypical heterogeneity are evidencing the need to find robust biomarkers as a key point towards their translation into the clinic, as well as better understanding the mechanisms involved in the induction of immune tolerance. With that aim, in this study we have compared the transcriptomic profile of tolDC induced with either vitamin D3 (vitD3-tolDC), dexamethasone (dexa-tolDC) or rapamycin (rapa-tolDC) through a microarray analysis in 5 healthy donors. The results evidenced that common differentially expressed genes could not be found for the three different tolDC protocols. However, individually, *CYP24A1*, *MUCL1* and *MAP7* for vitD3-tolDC; *CD163*, *CCL18*, *C1QB* and *C1QC* for dexa-tolDC; and *CNGA1* and *CYP7B1* for rapa-tolDC, constituted good candidate biomarkers for each respective cellular product. In addition, a further gene set enrichment analysis of the data revealed that dexa-tolDC and vitD3-tolDC share several immune regulatory and anti-inflammatory pathways, while rapa-tolDC seem to be playing a totally different role towards tolerance induction through a strong immunosuppression of their cellular processes.

## Introduction

In the last decade, tolerogenic dendritic cells (tolDC) have become one of the most promising approaches for the treatment of immune-mediated disorders such as autoimmune diseases (i.e. type 1 diabetes, multiple sclerosis or rheumatoid arthritis), but also for allergies or transplant rejection. In a healthy organism, immature dendritic cells (iDC) are specialized antigen-capturing cells that, when exposed to a pro-inflammatory millieu, differentiate into mature dendritic cells (mDC) in order to orchestrate an immunogenic response against the potentially pathogen-related peptide they previously recognized, captured and presented. Autoimmune disorders are characterized by the loss of immune tolerance against determined self-peptides, thus causing a pathological response of the immune system that leads to different diseases depending on which antigen/s are equivocally attacked. In this context, the main advantage of potential tolDC-based therapies resides in their presumed role to restore the immune tolerance against self-peptides in an antigen-specific manner, acting only over the cause of the pathologic process without compromising the protective immunity from the patient.

A wide variety of protocols has been developed to generate tolDC *in vitro*, for instance by the action of several immunomodulatory agents (such as 1,25-dihydroxycholecalciferol, the active form of vitamin D3^1–4^, dexamethasone^3–6^ or rapamycin^3,4,7^), cytokines (IL-10^4,8^, IFN-β^4,9^) or by genetic engineering^10,11^ and, in all cases, they remain stable against maturation. Furthermore, the leap from the bench to the bedside has already been taken, there existing several clinical trials, either completed or ongoing, that have demonstrated the safety of autologous tolDC-based therapies in rheumatoid arthritis, type 1 diabetes and Crohn’s disease, while further studies to evaluate their actual efficacy are currently being developed^11–16^. However, the characteristics of these tolerogenic cells are heterogeneous depending on which protocol was used to differentiate them, presenting, for instance, variable phenotypical characteristics or producing different cytokines. For this reason, a wide range of analyses has to be carried out to characterize them. Currently, the most reliable evidence of the regulatory properties of tolDC comes given by functional assays. However, these tests normally take days and require the generation of control immunogenic conditions in parallel, which also translates into an increase in the cost of an already expensive production process due to the strict good manufacturing practice (GMP) conditions that are required to generate clinical grade tolDC. Therefore, the need for common pathways or strong biomarkers that could define the concept of tolerogenicity and unequivocally characterize tolDC is one of the pending questions to be answered, as they would help to better understand the molecular mechanisms of tolerance as well as saving time and money during the manufacturing of the cell products.

Vitamin D3, dexamethasone and rapamycin are three of the most widely used drugs to induce the differentiation of tolDC *in vitro*. Our previous studies have shown that vitamin D3-induced tolDC (vitD3-tolDC) and dexamethasone-induced tolDC (dexa-tolDC) generate cells with rather similar characteristics in terms of presenting a semi-mature phenotype, increased IL-10 secretion and reduced allogeneic T cell proliferation priming. In contrast, rapamycin-induced tolDC (rapa-tolDC) seemed to develop their tolerogenic role through regulatory T cell (Treg) induction, despite their mature phenotype and not secreting IL-10. In all cases, however, an allogeneic T cell proliferation suppression was observed, and the three tolDC types remained stable upon LPS re-stimulation^3^.

Consequently, provided the heterogenous characteristics of these cells, we performed a microarray analysis of vitD3-, dexa- and rapa-tolDC, differentiated from 5 healthy donors, in order to obtain their transcriptomic profile and look for common pathways and/or mechanisms of tolerance induction. Indeed, our hypothesis is that the identification and definition of these effector routes could provide useful biomarkers for the characterization of these cells, specially thinking of their application in future clinical trials, since they may be helpful to compare results in studies worldwide and thus accelerate the translation of tolDC-based therapies from the bench to the bedside.

## Results

### Gene expression analysis revealed two different transcriptomic profiles in tolDC

The preprocessing steps described in the methods section left 7864 probesets to be included in the analysis. In order to look for potential common biomarkers for the three tolDC conditions, the expression between each tolDC vs mDC, as well as between mDC vs iDC, was compared in cells differentiated from 5 healthy donors, using the linear models approach described in the methods section. Among them, an additional comparison was carried out between dexa-tolDC, rapa-tolDC and vitD3-tolDC versus both mDC and iDC, yielding a total of 1216 genes showing a statistically significant differential expression in at least one comparison (p-value < 0.01).

**Figure 1:**
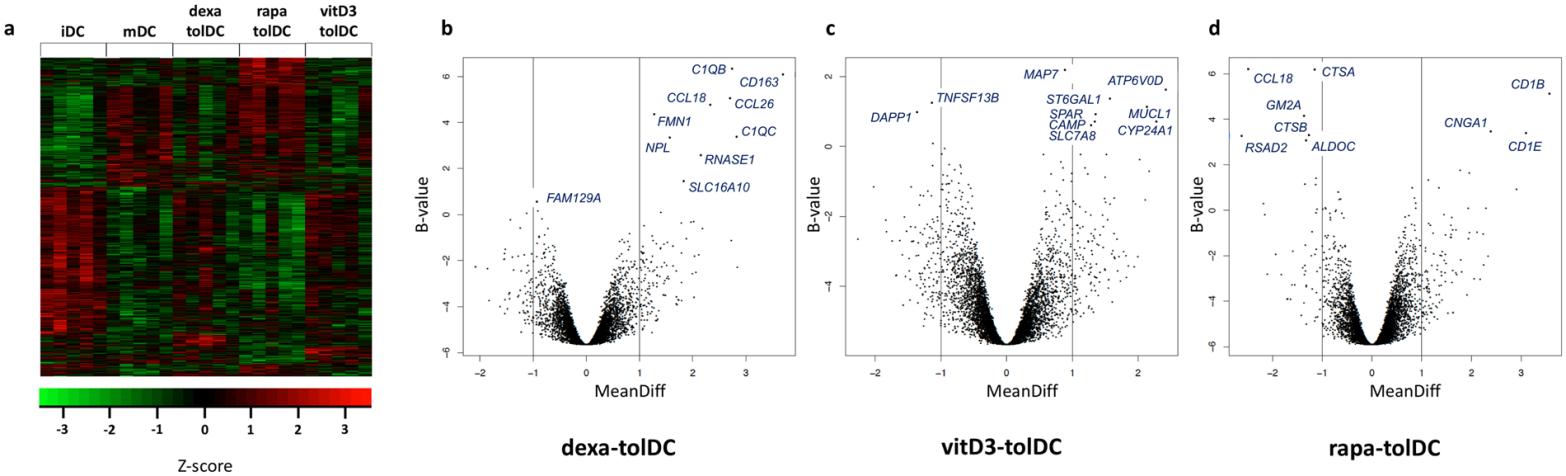
Comparative transcriptomic analysis of vitD3-tolDC, dexa-tolDC, rapa-tolDC, iDC and mDC. (**a**) Heat map representation of the transcriptomic expression profile of the different DC. Volcano plots of the top differentially expressed genes based on mean differences of expression (MeanDiff) and B scores of (**b**) dexa-tolDC, (**c**) vitD3-tolDC and (**d**) rapa-tolDC versus mDC.

The representation of the transcriptomic profile of the 5 types of DC (iDC, mDC, dexa-tolDC, rapa-tolDC and vitD3-tolDC) in a heat map evidenced a segregation into two clusters of 492 and 724 genes with opposed expression (Fig. 1a). As expected, iDC and mDC exhibited an opposed genetic signature. However, rapa-tolDC showed a similar profile to mDC, while vitD3- and dexa-tolDC presented more resemblance to iDC.

### TolDC showed several differentially expressed genes involved in the immune response modulation, signaling and trafficking compared to mDC

To find the genes involved in the tolerogenic function of vitD3-, dexa- and rapa-tolDC, the expression of each tolDC condition was compared with the expression of mDC. The results are presented as mean differences of the signals (MeanDiff) for each gene, and the B-statistic values were also considered. As a result, only those genes presenting B > 0 and p < 0.01 values were selected for each of the different tolDC conditions, as they would constitute the most relevant and likely candidates for being involved in tolerance.

When looking at the differentially expressed genes (DEG) between dexa-tolDC and mDC (Table 1), we found that there were several overexpressed genes (MeanDiff > 1.2) directly involved in immune-related functions such as the complement activation (*C1QB* and *C1QC*) and the immune-related chemotaxis (*CCL18* and *CCL26*), while others are mainly involved in metabolism and cell interaction. In contrast, only 3 down-regulated genes (MeanDiff < −0.6) presented a positive value for the B-statistic, *LSM14B* (which may play a role in mRNA translation), *FAM129A* (a regulator of p53-mediated apoptosis) and *PIWIL4* (involved in the development and maintenance of germline stem cells). The volcano-plot representation of the results can be observed in Fig. 1b.

**Table 1:** Differentially expressed genes in dexa-tolDC, vitD3-tolDC and rapa-tolDC versus mDC.

Gene	EntrezID	MeanDiff vs mDC	B-statistic	p-value	GO annotations
***dexa-tolDC***					
*CD163*	9332	3.70	6.08	<0.0001	Protein binding, scavenger receptor acivity
*C1QC*	714	2.83	3.39	<0.0001	Innate immune response, immune complement
*C1QB*	713	2.74	6.33	<0.0001	Innate immune response, immune complement
*CCL26*	10344	2.71	5.05	<0.0001	Chemotaxis, signal transduction, inflammatory response
*CCL18*	6362	2.34	4.79	<0.0001	Chemotaxis, signal transduction, inflammatory response
*RNASE1*	6035	2.16	2.59	<0.0001	Nucleic acid binding
*SLC16A10*	117247	1.83	1.45	0.0001	Amino acid transport
*NPL*	80896	1.57	3.37	<0.0001	Protein binding
*RGL1*	842953	1.40	0.11	0.0003	Protein binding
*FMN1*	342184	1.27	4.36	<0.0001	Microtubule cytoskeleton
*LSM14B*	149986	−0.91	0.15	0.0003	RNA binding
*FAM129A*	116496	−0.92	0.55	0.0002	Protein binding
*PIWIL4*	143689	−1.12	0.07	0.0004	RNA binding
***vitD3-tolDC***					
*ATP6V0D2*	245972	2.42	1.64	0.0001	Protein binding
*CYP24A1*	1591	2.27	0.72	0.0002	Metabolism
*MUCL1*	118430	2.13	1.13	0.0001	Metabolism
*ST6GAL1*	6480	1.56	1.38	0.0001	Metabolism
*CAMP*	820	1.35	0.71	0.0002	Innate immune response
*SPARC*	6678	1.35	0.93	0.0001	Protein binding
*SLC7A8*	23428	1.27	0.62	0.0002	Amino acid transport
*MAP7*	9053	0.88	2.23	<0.0001	Microtubule cytoskeleton
*GZMB*	3002	0.74	0.34	0.0003	Protein binding, immunological synapse
*PIWIL4*	143689	−1.12	0.09	0.0004	RNA binding
*TNFSF13B*	10673	−1.15	1.25	0.0001	Protein binding
*DAPP1*	27071	−1.37	0.99	0.0001	Protein binding
***rapa-tolDC***					
*CD1B*	910	3.56	5.12	<0.0001	Adaptive immune response
*CD1E*	913	3.09	3.38	<0.0001	Adaptive immune response
*CNGA1*	1259	2.40	3.46	<0.0001	Protein binding, plasma membrane
*CD1C*	911	2.29	0.09	0.0004	Adaptive immune response
*CYP7B1*	9420	1.95	1.63	0.0001	Oxidation-reduction process
*LOC100128175*	100128175	1.77	1.76	0.0001	N/A
*KIAA1586*	57691	1.13	1.34	0.0001	Ligase activity
*SFMBT1*	51460	0.88	0.08	0.0004	Protein binding, negative regulation of transctiption
*FAM129A*	116496	0.88	0.31	0.0003	Protein binding
*PSIP1*	11168	0.81	0.99	0.0001	RNA binding
*PSAP*	5660	−0.63	0.49	0.0003	Lipid binding
*P4HB*	5034	−0.66	0.48	0.0003	Metabolism
*FTL*	2512	−0.68	0.80	0.0002	Protein binding
*FAS*	355	−0.72	0.01	0.0005	Cell death induction
*RRAGD*	58528	−0.93	0.07	0.0004	Protein binding
*SOAT1*	6646	−0.99	0.05	0.0004	Protein binding
*SERINC2*	347735	−1.10	0.05	0.0004	Metabolism
*SCD*	6319	−1.12	1.40	0.0001	Oxidation-reduction process
*TPP1*	1200	−1.13	1.13	0.0001	Protein binding
*CTSA*	5476	−1.14	6.20	<0.0001	Protein metabolism
*CTSB*	1508	−1.26	3.32	<0.0001	Protein metabolism
*ALDOC*	230	−1.32	3.07	<0.0001	Protein binding
*CTSD*	1509	−1.35	1.14	0.0001	Protein metabolism
*GM2A*	2760	−1.37	4.19	<0.0001	Metabolism
*GPNMB*	10457	−2.18	0.30	0.0003	Protein binding
*CCL18*	6362	−2.47	6.23	<0.0001	Chemotaxis, signal transduction, inflammatory response
*RSAD2*	91543	−2.62	3.27	<0.0001	Protein binding

Results shown as mean difference of expression (MeanDiff). In all cases, B > 0 and p < 0.01. GO: Gene Ontology.

In the case of vitD3-tolDC, the up- and down-regulated genes compared to mDC were not so directly related to immune functions (Table 1). Metabolism, as well as cell differentiation, structure and signaling, were the most predominant related functions, with genes such as *MAP7*, *MUCL1* or *SPARC* strongly up-regulated (MeanDiff > 0.7). Nevertheless, genes encoding antimicrobial proteins (*GZMB* and *CAMP*) and proteins related with the direct metabolism of vitamin D3 (*CYP24A1*) could also be found, making a total of 9 up-regulated genes with B > 0. Among the down-regulated genes, only 3 fulfilled our criteria in the microarray, once again *PIWIL4* (demonstrating certain similarity between vitD3-tolDC and dexa-tolDC), *TNFSF13B* and *DAPP1* (both outstanding for being involved in immune regulation). All three of them showed strong reductions on their expression (MeanDiff < −0.6). The volcano-plot representation of the data is shown in Fig. 1c.

As for rapa-tolDC, as shown in Table 1, a total of 27 genes were selected. We found 3 genes with a strong up-regulation (MeanDiff > 2.2), encoding proteins developing innate immunity-related functions (*CD1B*, *CD1C* and *CD1E*), as well as, surprisingly, 2 genes related with the metabolism of fat soluble vitamins such as vitamin D3 (*CNGA1* and *CYP7B1*). Among the down-regulated genes, most of them were related with the metabolism of different molecules and proteins, especially outstanding *CTSB*, *ALDOC* and *GM2A* for their high B values (>3) and their strong down-modulation (MeanDiff < −1.2). The down-modulation of *FAS* gene, mediating the induction of cell death, was also relevant. Analogously, a volcano-plot representation of the results in rapa-tolDC is shown in Fig. 1d.

### A common genetic biomarker could not be found for the three tolDC conditions

Provided that a biomarker should unequivocally characterize a determined biological condition, we restricted even more our filtering parameters, selecting only those genes that were differentially expressed in the tolDC conditions versus both iDC and mDC at the same time. Once again, we made use of the P and B-statistic values as filtering criteria, selecting only those genes presenting B > 0 and p < 0.01 values for both comparisons. Consequently, we obtained those DEG that not only appeared to be differentially expressed, either over- or down-regulated, but that also their differential expression had high enough odds of being reliable.

**Figure 2:**
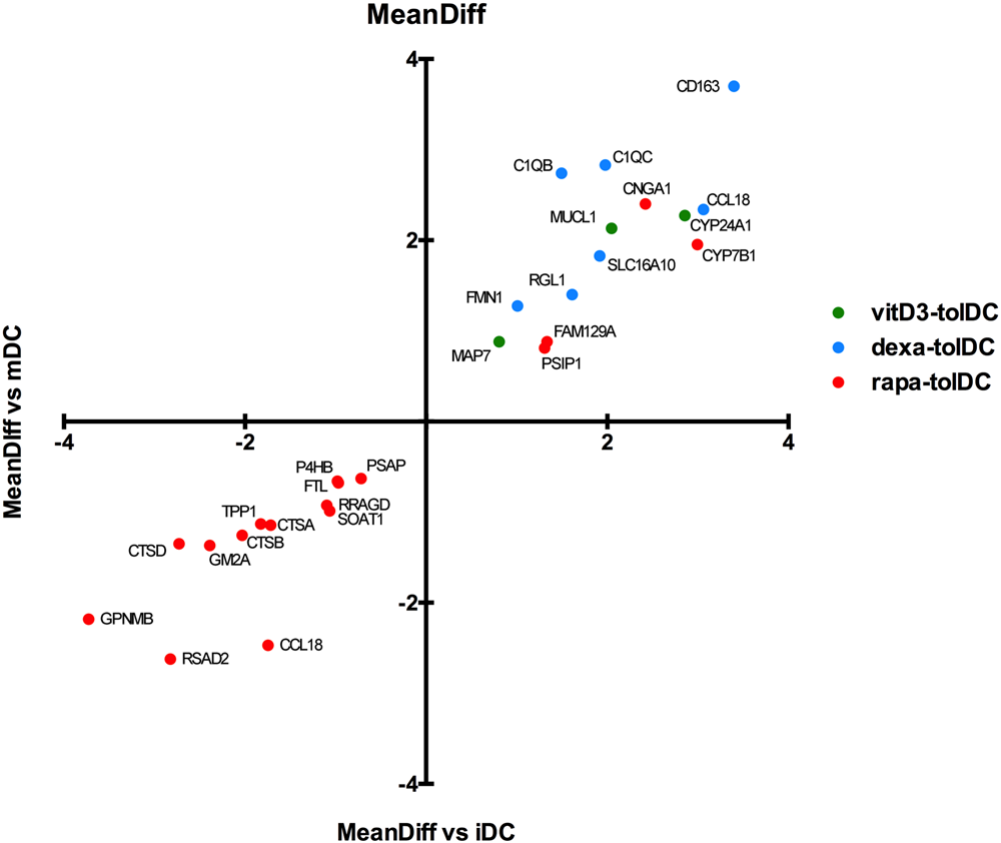
Differentially expressed genes in vitD3-, dexa- or rapa-tolDC versus both iDC and mDC with a B-statistic value > 0. Results shown as mean difference of expression (MeanDiff).

As a result, 26 different genes, many of them already mentioned in the previous section, were compliant with the filtering parameters in at least one tolDC condition; 3 of them were overexpressed in vitD3-tolDC, 7 genes in dexa-tolDC and, in the case of rapa-tolDC, 4 genes were up-regulated and 13 were down-regulated (Fig. 2). Among all those genes, only *CCL18* appeared in 2 out of the 3 tolDC conditions, showing a MeanDiff > 2.30 in dexa-tolDC but a MeanDiff < −1.69 in rapa-tolDC (p-value < 0.01). As for the other reported genes, many of them were related with immune functions or cell differentiation, interaction or signaling mechanisms, such as *MUCL1*, *MAP7*, *CD163*, *C1QB* or *C1QC*, indicating important changes in the status of the different tolDC conditions respect of iDC and mDC that might be relevant for the tolerogenic function of the cells, or simply induced by the different tolerogenic agents used. These genes presented at least a MeanDiff > 0.79 for the up-regulated ones and a MeanDiff < −0.60 for those down-regulated. In all cases, statistical significance was reached (p < 0.01). These and further details can be found in Table 2.

**Table 2:** Differentially expressed genes in vitD3-tolDC, dexa-tolDC and rapa-tolDC versus both mDC and iDC.

Cell type	Gene	EntrezID	Coding protein	*MeanDiff*		*B-statistic*		*p-value*	
				vs iDC	vs mDC	vs iDC	vs mDC	vs iDC	vs mDC
vitD3-tolDC	*CYP24A1*	1591	Vitamin D3 24-Hydroxylase	2.86	2.27	3.54	0.72	<0.0001	0.0002
	*MUCL1*	118430	Mucin-Like Protein 1	2.05	2.13	0.97	1.13	0.0002	0.0001
	*MAP7*	9053	Microtubule Associated Protein 7	0.80	0.88	1.60	2.23	0.0001	<0.0001
dexa-tolDC	*CD163*	9332	Cluster of Differentiation 163	3.40	3.70	6.06	6.08	<0.0001	<0.0001
	*C1QC*	714	Complement C1q C Chain	1.98	2.83	0.01	3.39	0.0005	<0.0001
	*C1QB*	713	Complement C1q B Chain	1.50	2.74	0.04	6.33	0.0004	<0.0001
	*CCL18*	6362	C-C Motif Chemokine Ligand 18	3.06	2.34	9.83	4.79	<0.0001	<0.0001
	*SLC16A10*	117247	Solute Carrier Family 16 Member 10	1.92	1.83	2.37	1.45	<0.0001	0.0001
	*RGL1*	842953	RalGDS-Like 1	1.61	1.40	1.76	0.11	0.0001	0.0003
	*FMN1*	342184	Formin 1	1.01	1.27	2.21	4.36	<0.0001	<0.0001
rapa-tolDC	*CNGA1*	1259	Cyclic Nucleotide Gated Channel Alpha 1	2.42	2.40	3.77	3.46	<0.0001	<0.0001
	*CYP7B1*	9420	Oxysterol 7-Alpha-Hydroxylase	3.00	1.95	7.67	1.63	<0.0001	0.0001
	*FAM129A*	116496	Cell Growth-Inhibiting Gene 39 Protein	1.34	0.88	5.46	0.31	<0.0001	0.0003
	*PSIP1*	11168	PC4 And SFRS1 Interacting Protein 1	1.31	0.81	7.49	0.99	<0.0001	0.0001
	*PSAP*	5660	Prosaposin	−0.72	−0.63	1.88	0.49	0.0001	0.0003
	*P4HB*	5034	Prolyl 4-Hydroxylase Subunit Beta	−0.98	−0.66	5.45	0.48	<0.0001	0.0003
	*FTL*	2512	Ferritin Light Chain	−0.97	−0.68	5.35	0.80	<0.0001	0.0002
	*RRAGD*	58528	Ras Related GTP Binding D	−1.10	−0.93	1.79	0.07	0.0001	0.0004
	*SOAT1*	6646	Sterol O-Acyltransferase 1	−1.07	−0.99	0.72	0.05	0.0002	0.0004
	*TPP1*	1200	Tripeptidyl Peptidase 1	−1.82	−1.13	7.71	1.13	<0.0001	0.0001
	*CTSA*	5476	Cathepsin A	−1.72	−1.14	13.71	6.20	<0.0001	<0.0001
	*CTSB*	1508	Cathepsin B	−2.03	−1.26	11.08	3.32	<0.0001	<0.0001
	*CTSD*	1509	Cathepsin D	−2.73	−1.35	11.59	1.14	<0.0001	0.0001
	*GM2A*	2760	GM2 Ganglioside Activator	−2.39	−1.37	13.72	4.19	<0.0001	<0.0001
	*GPNMB*	10457	Glycoprotein NMB	−3.73	−2.18	7.27	0.30	<0.0001	0.0003
	*CCL18*	6362	C-C Motif Chemokine Ligand 18	−1.75	−2.47	1.94	6.23	0.0001	<0.0001
	*RSAD2*	91543	Viperin	−2.82	−2.62	4.48	3.27	<0.0001	<0.0001

Results shown as mean difference of expression (MeanDiff). In all cases, B > 0 and p < 0.01.

### VitD3 and dexa-tolDC share several common regulatory pathways, although none of them with rapa-tolDC

**Figure 3:**
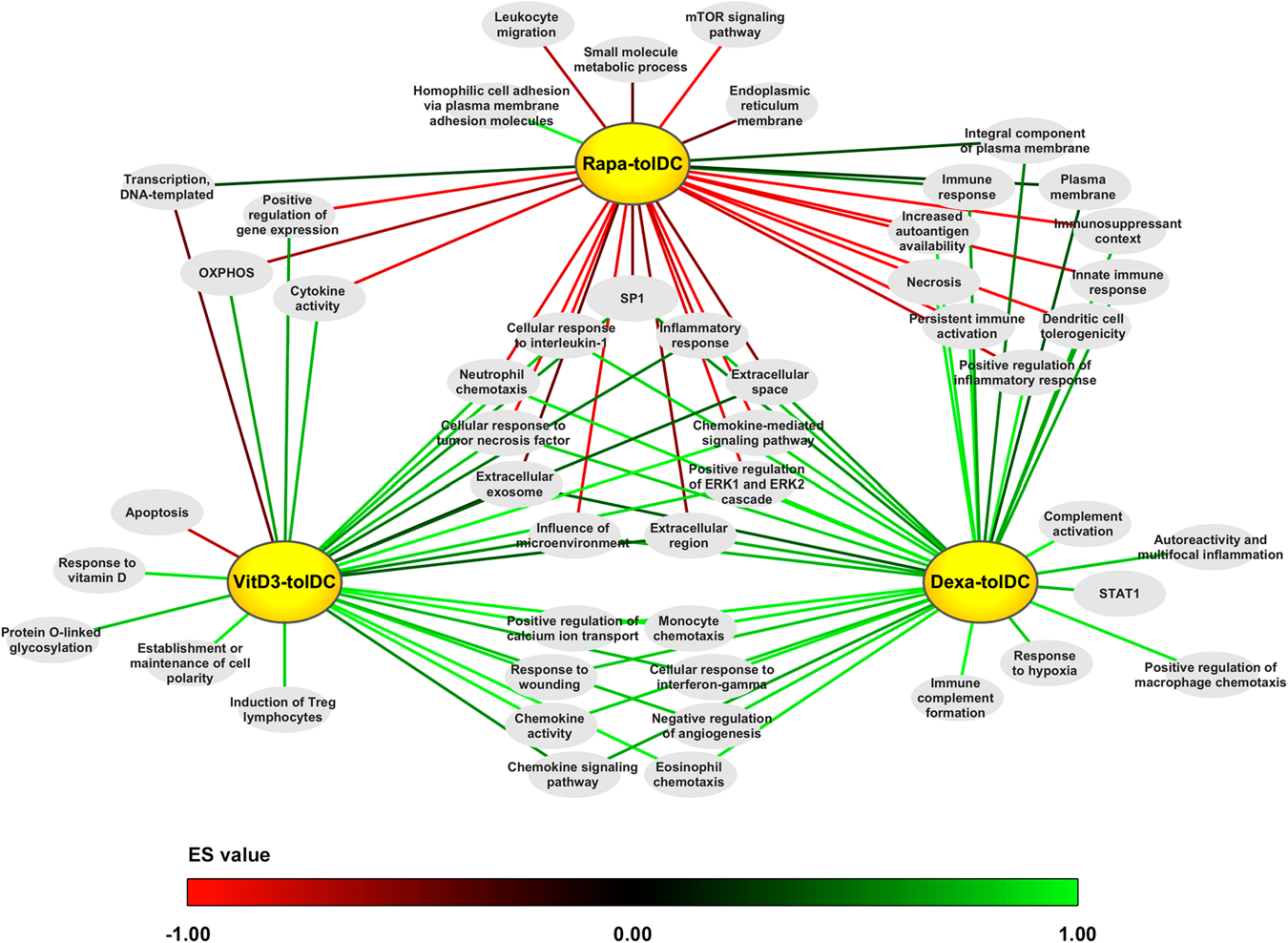
Graphical representation of the enriched pathways and protein sets in vitD3-, dexa- and/or rapa-tolDC. The color code indicates the degree of enrichment of each protein set based on their ES, from red (ES = −1) to green (ES = 1) as indicated by the color scale bar.

After determining which DEG could be found on each condition, we decided to perform a more comprehensive study of the transcriptome by analyzing which pathways and protein sets were up- or down-modulated on each DC condition. To do this, a Gene Set Enrichment Analysis (GSEA) was performed, and only those pathways and protein sets that showed a statistically significant enrichment (p-value < 0.05) on each tolDC condition compared to mDC were considered. Additionally, all those pathways that were up-modulated on iDC versus mDC were excluded as they would not constitute differential pathways of tolerance for our tolDC products, with the exception of the induction of Treg lymphocytes, immune response and hemophilic cell adhesion via plasma membrane adhesion molecules protein sets, due to their functional relevance in tolerance. Finally, a total of 49 pathways and protein sets, differentially expressed versus mDC, were selected, either due to their relevance or for being shared between at least 2 tolDC conditions (Table 3). A graphical representation of them is presented in Fig. 3. The analysis could not reveal any pathway up- or down-modulated in common between the three tolDC conditions versus mDC.

**Table 3:**
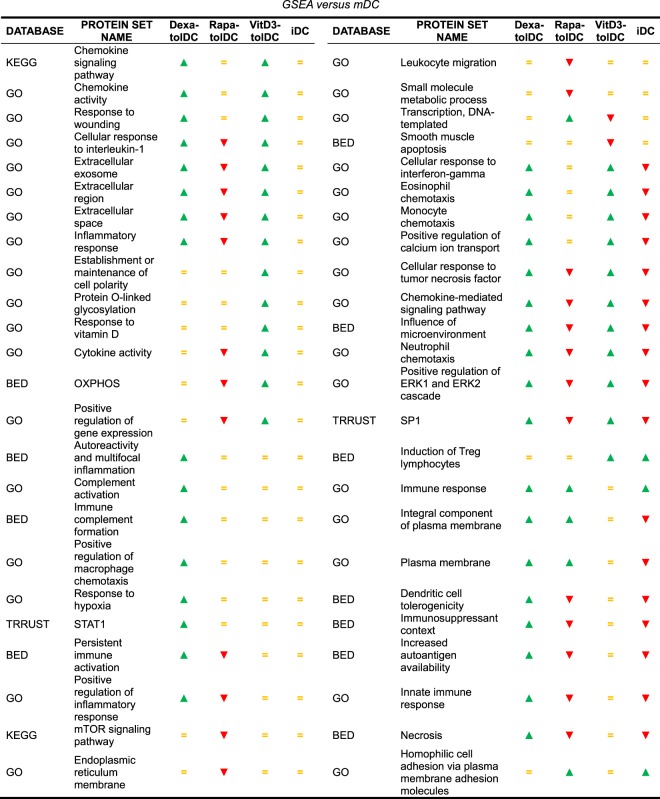
Enriched protein sets in dexa-tolDC, rapa-tolDC, vitD3-tolDC and/or iDC versus mDC. Green arrow: upregulation of said set; Yellow bar: unchanged regulation of said set; Red arrow: downregulation of said set. BED: Biological Effectors Database; GO: Gene Ontology; KEGG: Kyoto Encyclopedia of Genes and Genomes; TRRUST: Transcriptional Regulatory Relationships Unraveled by Sentence-based Text-mining.

When taking the comparisons two by two, a total of 18 pathways were simultaneously up-regulated in both dexa and vitD3-tolDC versus mDC, and 3 protein sets, mainly related with the plasma membrane, appeared up-regulated in dexa and rapa-tolDC versus mDC, with different behaviors regarding the comparisons between the remaining conditions. Any common enriched protein sets could be found between vitD3- and rapa-tolDC. Further 13 pathways were enriched at the same time in rapa-tolDC and either dexa or vitD3-tolDC, but with opposite modulation. Among these 34 mentioned protein sets, only 8 were differentially induced versus mDC in at least two tolDC conditions, being them dexa- and vitD3-tolDC in all cases, and with no differences between iDC and mDC. In addition, in 5 of those cases, the protein sets were also simultaneously down-modulated in rapa-tolDC. Of them, 3 were related with extracellular components (extracellular region, extracellular space and extracellular exosome) and the other 2 with a response to inflammation stimuli (inflammatory response and cellular response to IL-1).

Additionally, a GSEA was also performed comparing tolDC and mDC versus iDC, and in this case both nucleosome assembly and autoreactivity and multifocal inflammation protein sets were found differentially overexpressed only in dexa, rapa and vitD3-tolDC at the same time, as there were no differences in their expression between iDC and mDC. However, again, the comparison of any pathway or biomarker in tolDC versus iDC could potentially provide results caused by the maturation process that these cells were exposed to, just like mDC, and not exclusively by the tolerogenic features of the cells. In fact, these same two protein sets did not show the same pattern in the previous GSEA versus mDC. Consequently, those sets which were upregulated in mDC versus iDC were excluded from the analysis. The results can be seen in Supplementary Table S1.

### VitD3-tolDC presented an increased metabolic activity combined with a reduction in the apoptotic processes

When considering the pathways simultaneously regulated in vitD3-tolDC versus both iDC and mDC, we encountered that, as expected, those related with oxidative phosphorylation and the metabolism of vitamin D3 were overexpressed. In addition, the protein O-linked glycosylation pathway was also found overexpressed in vitD3-tolDC in comparison to mDC, as already reported in previous studies^17,18^. Furthermore, the ERK1/2 signaling cascade and the SP1 signaling factor, both involved in important tolerogenic functions, were induced in vitD3-tolDC respect of mDC. Consequently, the tolerance-inducing functionality of vitD3-tolDC is suggested to be driven by the up-regulation of the Treg lymphocyte induction genes and an increased expression of the extracellular region protein set compared to both iDC and mDC, together with the results shown in the previous section. These protein sets contain, in fact, important immune-related genes such as *CCL4* and *CCL7*, which determine T cell and monocyte chemotaxis respectively, as well as *MUCL1*, previously mentioned as a potential biomarker. Other up-regulated protein sets included viral and inflammatory response activities. In contrast, only the apoptosis pathway appeared to be differentially down-regulated in vitD3-tolDC. All the results are presented in Table 3 and Table 4.

**Table 4:**
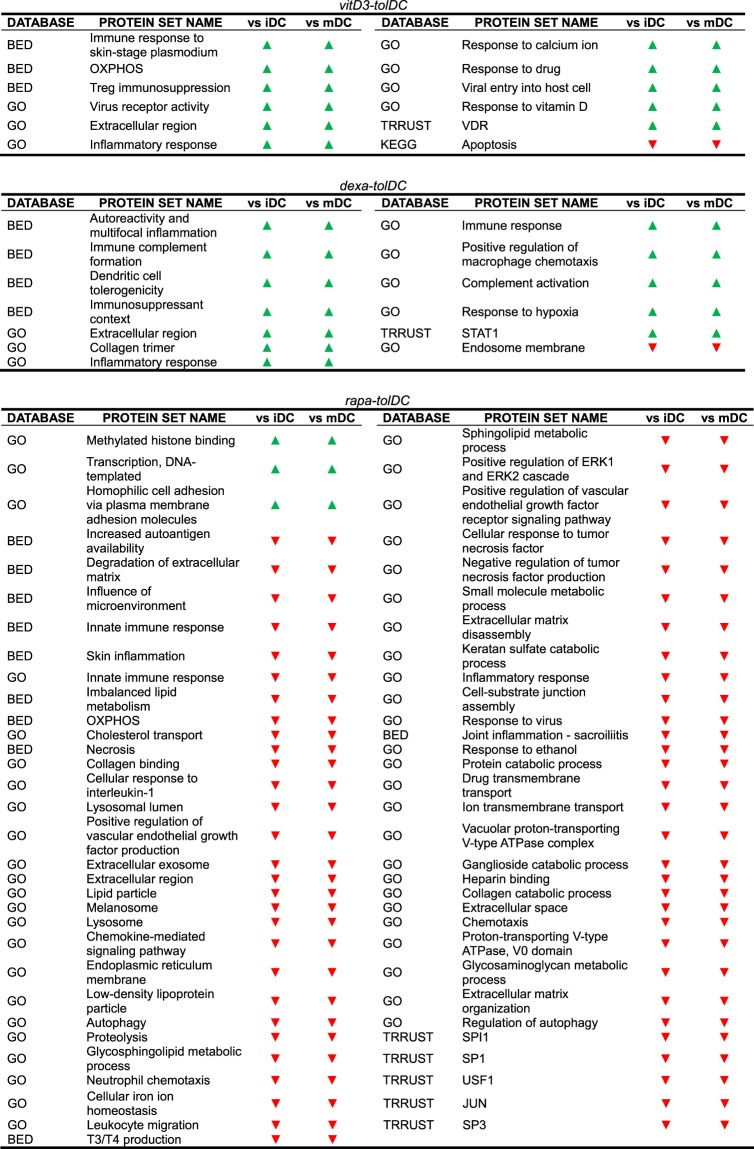
Enriched pathways and protein sets, versus both iDC and mDC, in vitD3-tolDC, rapa-tolDC and dexa-tolDC. Green arrow: upregulation of said set; Yellow bar: unchanged regulation of said set; Red arrow: downregulation of said set. BED: Biological Effectors Database; GO: Gene Ontology; KEGG: Kyoto Encyclopedia of Genes and Genomes; TRRUST: Transcriptional Regulatory Relationships Unraveled by Sentence-based Text-mining.

### A strongly down-regulated transcriptome is observed in rapa-tolDC

Contrary to vitD3-tolDC, the transcriptomic profile of rapa-tolDC was mostly consisting on down-modulated protein sets, evidencing 64 pathways that were repressed in comparison with both iDC and mDC. Of note, many of these down-modulated pathways were related with inflammation, chemotaxis and lipid metabolism (Table 4). Among them, 11 of these protein sets were those that appeared simultaneously up-regulated in dexa- and vitD3-tolDC, as mentioned above. As for the rest, many signaling, metabolic and transportation processes were inhibited in rapa-tolDC, such as the ERK1 and ERK2 cascade or the glycosphingolipid metabolism and cholesterol transport. Moreover, several protein sets related with the inflammatory and innate immune responses were also found inhibited, evidencing the potent immunosuppressant effect of rapamycin. Confirming previous reports, and as therefore expected, the mTOR pathway also appeared down-modulated in rapa-tolDC referred to mDC but not to iDC^19–21^ (Table 3). On the other hand, only 3 protein sets were upregulated, being especially relevant the methylated histone binding and the DNA-templated transcription, as they indicate that deep changes might be happening regarding the DNA processing and epigenetics of rapa-tolDC.

### Immune complement and macrophage features are expressed in dexa-tolDC

Similarly to vitD3-tolDC, dexa-tolDC presented a mostly up-regulated differential transcriptomic profile (Table 4). However, the induction of immune-related protein sets was much more relevant in this condition, with the positive regulation of immune complement activation and macrophage chemotaxis pathways. In addition, the up-modulation of immunosuppressant and DC tolerogenicity protein sets, along with the induction of the ERK1/2 signaling cascade and the SP1 transcription factor, supports the tolerogenic functionality of dexa-tolDC. Moreover, the increased expression of the extracellular region protein set was also directed towards the immune function, with *CCL2*, *CCL4, CD163* and several other immune-related protein-encoding genes up-regulated. However, STAT1 appeared to be up-modulated in dexa-tolDC, which constituted an unexpected result due to its generally pro-inflammatory-related functionality. Another similarity with previously reported results for vitD3-tolDC was the up-modulation of the response to hypoxia also in dexa-tolDC^17^.

## Discussion

The number of clinical trials using autologous tolDC to treat autoimmune diseases is increasing each year, and the first results from several phase I studies have demonstrated that this tolerogenic therapy is safe for the patients^11–16^. Therefore, the role of these cells is gaining a huge relevance in the field of personalized medicine. Due to the wide variety of protocols that exist nowadays to generate tolDC *in vitro*, a deep study of the cells generated by them has become of key importance to elucidate which mechanisms of tolerance induction are being triggered. Establishing adequate quality controls and biomarkers that can ensure not only the functionality but also the safety of tolDC has become one of the main concerns towards its translation into the clinic^22^. Thus, determining if common pathways of tolerance are being promoted or whether each treatment is activating different mechanisms in the cellular product is important, as it would set up the first steps towards the finding of potential biomarkers of tolDC. Ideally, however, they should be able to generically identify these cells despite the protocol used to generate them.

To our knowledge, our microarray analysis constitutes the first study directly comparing three of the most widely used tolDC-inducing protocols. Unfortunately, it was not possible to find a common DEG in the transcriptomic profile of vitD3-, dexa- and rapa-tolDC. In fact, just a brief analysis of the whole transcriptomic profile looking at the heat map already evidenced that different protocols came with different prints, as rapa-tolDC showed not only a different but a completely opposite genetic signature compared to dexa- and vitD3-tolDC. These results are in accordance with a previous study by our group that evidenced different phenotypical and functional characteristics of dexa, rapa and vitD3-tolDC^3^. Our current study allowed us to go deeper in that direction and, in fact, we could identify some potential biomarkers for both rapa- and dexa-tolDC, *CCL18* and *FAM129A* genes. However, they showed an opposed behavior pattern –while *CCL18* appeared to be differentially induced in dexa-tolDC, it was down-modulated in rapa-tolDC, and vice versa for *FAM129A*–, evidencing that the molecular tolerance-inducing mechanisms triggered by these two immunomodulatory agents must be different. Specifically, *CCL18* is of a notorious relevance, since the cytokine encoded by this gene has been reported to have a fundamental role in the cell differentiation process towards the development of semi-mature DC with the ability to produce IL-10 and prime Treg^23^.

The fact that common candidate biomarkers could not be found for our three tolDC-inducing protocols, however, does not decrease the value of those genes that appeared as differentially expressed on each one of the studied conditions versus both iDC and mDC. For instance, as indicators of the up-regulation of the protein O-linked glycosylation, of the response to vitamin D treatment, and of the establishment of cell polarity, detected by the GSEA in vitD3-tolDC, *MUCL1*, *CYP24A1*, and *MAP7* genes, respectively, were found strongly induced versus iDC and mDC at the same time. Provided that the O-linked glycosylation has been directly related with the regulation of microtubule-associated proteins of the cytoskeleton^24^, combined with previous studies reporting *CYP24A1* and *CAMP* genes as directly related to the vitamin D3 metabolism^25–28^, all these genes apparently make good candidates to become strong biomarkers of vitD3-tolDC. Additionally, these candidates have also been related to many other key cell processes such as the glucose metabolism, stress response and cell cycle, as reviewed by Hart *et al*.^29^. Moreover, their discovery is also supported by the positive B-statistic values showed by these genes in the microarray. Further supporting our results, an induction of the oxidative metabolism could also be detected, which constitutes a key feature of vitD3-tolDC, as previously reported^17^.

In the case of dexa-tolDC, apart from *CCL18*, already discussed above, the overexpression of the genes encoding CD163 and two different chains of the complement C1q protein (*C1QB* and *C1QC*) versus both iDC and mDC are the most relevant results as potential biomarkers, due to their immune-related implications. It is also worth mentioning that *MERTK* has been previously reported as a biomarker for dexa-tolDC in several studies^6,30^. Our results, however, do not evidence the differential expression of this gene, but it might be explained due to the intrinsic limitations of microarrays regarding false negatives results. As for *CD163*, its role in tolerance induction has already been reported in M2 macrophages but, so far, not for tolDC. We have also detected an enrichment on the macrophage chemotaxis protein set. Therefore, our results suggest that both regulatory macrophages and dexa-tolDC might be triggering similar tolerogenic mechanisms, probably through the STAT3 and Wnt5a signaling pathway, as its interaction with CD163 has already been reported in cancer studies^31,32^. Regarding *C1QB* and *C1QC* genes, their overexpression is aligned with the up-modulation of the complement activation protein set. The role of the immune complement system, as a promoter of immune tolerance in dendritic cells, has been overlooked until the last few years. Nevertheless, as reviewed by Luque *et al*.^33^, recent studies demonstrate that C1q is involved in key tolerogenic processes such as an increased surface PD-L2 and decreased CD86 expression, linked to a reduced induction of Th1 and Th17 proliferation^34^, the inhibition of the production of pro-inflammatory mediators^35^ and an increased production of anti-inflammatory cytokines, such as IL-10^36,37^. As a matter of fact, *C1QB* has previously been proposed as a potential biomarker for tolerogenicity^38^, and its differential expression along with other genes encoding the C1q protein has been reported in previous studies^30^. Therefore, our results seem to indicate that dexa-tolDC might be developing their tolerogenic properties through the mentioned mechanisms, among others that will be discussed below. Conversely, our microarray also detected an induction of the STAT1 signaling pathway, which has been reported as pro-inflammatory and opposed to STAT3, switching between both responses under the control of NOD1 after an IL-10-dependant activation^39^. However, other studies also reported that, in regulatory dendritic cells such as dexa-tolDC, STAT1 can be activated in response to TLR stimuli in order to attract Th1 cells through an increased CXCL10 production and subsequently inhibit them^40^.

Comparative studies between vitD3- and dexa-tolDC have been reported before, demonstrating many similarities between the two conditions regarding their semi-mature status and the inhibition of allogeneic proliferation^3^, the NF-κB pathway suppression^41–43^ and the polarization of the immune response towards a Th2 profile^44^. Some differences, however, have been described regarding the antigen-specific induction of Treg^45^, and a proteomic comparative study also evidenced differences in the protein expression profile, despite confirming that vitD3- and dexa-tolDC were very similar on the phenotypical and functional aspects^46^. Furthermore, most of the mentioned studies also evidenced that the effect of both drugs was syngeneic, enhancing the tolDC-inducing effect of vitamin D3 and dexamethasone when used in combination, instead of each one independently. In fact, this approach has even been tested on a clinical trial, with successful results regarding the tolerability and safety of the cell product^16,47,48^. Our study confirmed this resemblance between vitD3- and dexa-tolDC, as well as their tolerogenic potential, since a strong up-modulation of the ERK1/2 and SP1 pathways was observed in both conditions, among other protein sets. These specific pathways have been reported to be involved in key mechanisms of tolerance induction, such as, TGF-ß secretion^49–51^, dendritic cell survival^52^, TLR-dependent and independent IL-10 production^51,53,54^, and functional stability^55^. Surprisingly, however, we could not find any DEG in common for both dexa- and vitD3-tolDC respect of mDC, despite sharing the induction of such key pathways.

In addition, our results also showed that not only rapa-tolDC do not share the up-regulation of any of the discussed pathways in common with the other studied tolDC conditions, but that they are even down-modulated after rapamycin treatment. Furthermore, mTOR signaling has been reported as a crucial and even indispensable mechanism to maintain the tolerogenic functionality of vitD3- and dexa-tolDC in some of the same reports cited above^17,55^. Therefore, and provided that rapamycin is, indeed, the natural inhibitor of the mTOR signaling pathway, the transcriptomic and functional incompatibility of both dexa- and vitD3-tolDC with rapa-tolDC becomes evident. Consequently, our results suggest that different mechanisms might be triggered in rapa-tolDC to induce immune tolerance.

The down-modulation of the mTOR signaling by the response to rapamycin constitutes the main signature of these cells, and through the inhibition of its dependent pathways, several immune-related mechanisms have been reported to play a role in the induction of tolerance, as reviewed by Stallone *et al*.^56^. For instance, rapamycin has been described to both induce the up-regulation of CCR7 and dampen the production of IL-10 in monocyte-derived DC, but also that the surface expression of the former is inhibited by the latter^57^. Furthermore, the rapamycin-mediated inhibition of mTOR also reportedly induces the expression of ILT3 and ILT4 in DC, through the down-modulation of CD40, in order to prime Foxp3^+^ Treg and switch the immune response towards a Th2 profile^58^. Consequently, and in accordance to our results, the low IL-10 secretion by rapa-tolDC is functionally logical and demonstrates that tolerance can be achieved by different mechanisms that look apparently contradictory at first sight. Apart from the inhibition of mTOR, the effect of rapamycin comes along with the repression of many other immune-related genes, pathways and proteins. Many of them are involved in pro-inflammatory and chemotactic processes, thus demonstrating the strong immunosuppressant effect of this drug. In fact, while only overexpressed genes could be detected as potential biomarkers in the case of vitD3- and dexa-tolDC, for rapa-tolDC, from a total of 17 selected DEG, 13 of them were repressed and only 4 appeared up-modulated respect both iDC and mDC. A similar situation was evidenced for the selected protein sets after the GSEA analysis, both exclusively and in comparison to the other tolDC conditions, as discussed above.

In any case, the incapability to find common biomarkers arises the idea that, although a normalized transcriptomic profile of immune tolerance induction might not be achieved, at least a small pool of the most representative genes of each condition, constituting a “generic” tolDC signature, could be established. Nevertheless, it is worth stating that single results obtained from microarrays are highly prone to be biased, as the generally low B-statistic values found in our results suggest. Therefore, we cannot fully discard the possibility of having overlooked a determined universal genetic biomarker of tolerance, just like we did, for instance, with MERTK in dexa-tolDC. Nevertheless, this scenario seems unlikely given the strong differences that we have observed among the transcriptomic profiles of our tolDC conditions, and that were confirmed by the GSEA. Indeed, enrichment analyses provide an increased reliability to microarray studies, as they are based in the grouped expression of genes instead of single results and, as a matter of fact, many of the genes and pathways found in our array for each individual tolDC protocol have been previously reported and even evidenced in similar transcriptomic and proteomic studies^7,17,30,46,59^, thus strengthening our results.

In conclusion, and despite further validation is required, *CYP24A1*, *MUCL1*, *MAP7*, *CD163*, *CCL18*, *C1QB*, *C1QC*, *CYP7B1* and *CNGA1* genes, among several others, have been identified as potential biomarkers for the different individual tolDC-generating protocols. Furthermore, we have also been able to identify several pathways that are being differentially modulated by the pharmacological tolDC-inducing treatments, suggesting that immune tolerance is a complex status that can be achieved through different mechanisms. After all, several publications have demonstrated the capability of these protocols to generate functional immune regulatory cells, despite their differences. This functional heterogenicity, however, also suggest that determined tolDC-inducing protocols might be more suitable than others for the treatment of specific autoimmune diseases. For instance, a defect on the functionality and activation of Treg has been described in patients with type 1 diabetes and myasthenia gravis^60–62^. Consequently, based on both the literature and our current and previous results^3,45,56,58^, vitD3-tolDC and rapa-tolDC might constitute better therapeutic alternatives than dexa-tolDC in these two specific examples, since the induction of Treg plays an important role in their tolerogenic functionality. On the other hand, in diseases in which the presence of autoreactive T cells plays a main role, such as multiple sclerosis^63,64^, the vitD3-tolDC-mediated induction of hyporesponsiveness over these pathologic cells might have a more beneficial effect. However, this is far from demonstrated yet and, provided the complexity of the mechanisms of tolerance induction within the immune system, several *in vitro* experiments and clinical trials should be conducted in order to compare the efficacy of different protocols. In any case, and although our results seem to indicate that finding a common biomarker of tolerogenicity might be utopic, they also reinforce the role of tolDC as a promising therapeutic approach for the immediate future.

## Methods

### Sample collection and *in vitro* tolDC generation

Five samples from healthy donors of iDC, mature mDC and the three conditions of tolDC differentiated in the presence of either vitamin D3 (vitD3-tolDC), dexamethasone (dexa-tolDC) or rapamycin (rapa-tolDC) were selected from previous experiments by our group^3^. The Ethical Committee of Germans Trias i Pujol Hospital approved the study, and all subjects gave their informed consent according to the Declaration of Helsinki (BMJ 1991; 302: 1994). Briefly, for the DC differentiations, buffy coats provided by the *Banc de Sang i Teixits* (Barcelona, Spain) were processed, first depleting T CD3^+^ cells using a RosetteSep Human CD3 Depletion Cocktail (StemCell Technologies, Vancouver, Canada) during a ficoll-hypaque (Rafer, Zaragoza, Spain) gradient separation and later isolating monocytes by positive selection using the EasySep Human CD14 Positive Selection Kit (StemCell Technologies). In all cases, purity was greater than 95% and viability greater than 90%. Monocytes were cultured for 6 days in cGMP-grade X-VIVO 15 medium, supplemented with 100 U/mL penicillin and 100 µg/mL streptomycin, in the presence of 1000 U/mL clinical-grade granulocyte-macrophage colony- stimulating factor (GM-CSF; CellGenix, Freiburg, Germany) and 1000 U/mL clinical-grade interleukin 4 (IL-4; CellGenix). Respectively, half and total volume of fresh medium and cytokines were replenished on days 2 and 4. All the conditions except for iDC were treated on day 4 with a maturation cocktail of clinical-grade cytokines containing 1000 U/mL tumor necrosis factor alpha (TNFα; CellGenix), 10 ng/mL IL-1β (CellGenix) and 1 µM prostaglandin E2 (PGE2; Pfizer, New York, NY, USA). While mDC did not receive any additional stimulus, the different tolDC conditions were obtained adding either 1 nM vitamin D3 (Calcijex, Abbott, Chicago, IL, USA) on days 0 and 4, 1 µM dexamethasone (Fortecortín, Merck, Spain) on days 2 and 4 or 10 nM rapamycin (Rapamune, Wyeth, Spain) on days 2 and 4. In order to determine optimal and comparable concentrations of each of these immunomodulatory agents, dose-dependent experiments were set up using mDC as reference. Cells were harvested on day 6 for further characterization and functional assays, and later centrifuged and stored as dry pellets at −80 °C. The complete characterization of vitD3-, dexa- and rapa-tolDC regarding phenotype, cytokine secretion and functionality can be found in our previous study by Naranjo-Gómez *et al*.^3^.

### Preparation of RNA samples and microarray analysis

Total RNA was isolated from the dry pellet samples using RNeasy Kit (QIAGEN, Hilden, Germany) according to the manufacturer’s instructions, and RNA Integrity Number (RIN) was assessed. Only samples with good quality were considered (RIN ≥ 6). Total RNA was later retrotranscribed, and the resulting cDNA was further preamplified using the Ovation® PicoSL WTA System V2 kit (NuGEN Technologies, San Carlos, CA, USA) at the *Unitat Cientificotècnica de Suport* of the Vall d’Hebron Research Institute (Barcelona, Spain), due to the low amount of RNA initially obtained in some of the samples (1–300 ng). Subsequently, the cDNA was fragmented, labeled and hybridized to the 33297 probes of a GeneChip 1.0 microarray chip (Affymetrix, Santa Clara, CA, USA). The statistical analysis was performed using R software and the libraries developed for microarray data analysis by the Bioconductor Project (www.bioconductor.org). All the samples demonstrated high quality cDNA characteristics, with a 3′/5′ ratio of probe sets for glyceraldehyde-3-phosphate dehydrogenase and beta-actin of <1.5.

### Differentially expressed genes selection

All the images generated by the microarray were processed at the Department of Statistics from the University of Barcelona. The raw data obtained from the image (“CEL”) files were pre-processed using the robust multi-array average method^65^, which performs a three-step process consisting of background correction, normalization and summarization at gene level. The resulting expression values were then submitted to a two-step non-specific filtering process; First, those genes whose mean signal per group was below the 50^th^ percentile of all signals were removed. From the remaining genes, those whose standard deviation was below the 50^th^ percentile of all standard deviations were further filtered out. These normalized filtered values were used for all the analysis. The selection of DEG was based on a linear model analysis with empirical Bayes moderation of the variance estimates, following the methodology developed by Smyth^66^. The Benjamini and Hochberg method^67^ was used to adjust the p-values in order to obtain a strong control over the false discovery rate. For each gene, B-statistic values were calculated. Briefly, this parameter roughly indicates the logarithm of the odds of a gene to be effectively differentially expressed, and the higher the B value, the more likely that one determined result is reliable.

### Identification of enriched pathways and protein sets

A GSEA was performed by Anaxomics (Barcelona, Spain) over our microarray data in order to determine the presence of enriched pathways and protein sets between our different tolDC conditions, following previously described methodology^68^. The analysis was performed over protein sets from several databases, including Gene Ontology (GO) terms (biological process, cellular component and molecular function) according to the European Molecular Biology Laboratory-European Bioinformatics Institute (EMBL-EBI)/UniProt-GO^69^, Biological Effectors Database (BED, property of Anaxomics), Kyoto Encyclopedia of Genes and Genomes (KEGG)^70^, Pharmacogenomics Knowledgebase (PharmGKB)^71^, Small Molecule Pathway Database (SMPDB)^72^ and the regulatory molecular mechanisms included in the Transcriptional Regulatory Relationships Unraveled by Sentence-based Text-mining (TRRUST) database^73^. The degree of enrichment of a determined protein set was evaluated based on their respective enrichment score (ES). Cytoscape 3.5.1. software was used to create the representation of the common and individual enriched protein sets between each tolDC condition, based on their ES score.

### Accession code

Microarray data have been deposited in the ArrayExpress database at EMBL-EBI (www.ebi.ac.uk/arrayexpress) under accession number E-MTAB-6937 (https://www.ebi.ac.uk/arrayexpress/experiments/E-MTAB-6937).

## Electronic supplementary material

Supplementary Table S1
