# Supplementary material for: Comparative transcriptomic profile of tolerogenic dendritic cells differentiated with vitamin D3, dexamethasone and rapamycin
Source: Sci Rep. 2018 Oct 8;8:14985. doi: 10.1038/s41598-018-33248-7 (PMC6175832; doi:10.1038/s41598-018-33248-7)
Supplement: Supplementary file 1 — Supplementary Table S1 [file 41598_2018_33248_MOESM1_ESM.docx]

**SUPPLEMENTARY INFORMATION**

**Comparative transcriptomic profile of tolerogenic dendritic cells differentiated with vitamin D3, dexamethasone and rapamycin**

**Juan Navarro-Barriuso**^1,2^**,** **María José Mansilla**^1,2^**, Mar Naranjo-Gómez**^1^**,**

**Alex Sánchez-Pla**^3^**, Bibiana Quirant-Sánchez**^1,2^**, Aina Teniente-Serra**^1,2^**, Cristina Ramo‑Tello**^4^**, Eva M. Martínez‑Cáceres**^1,2,*^

^1^ Germans Trias i Pujol University Hospital and Research Institute. Immunology Division. Badalona, 08916, Spain

^2^ Universitat Autònoma de Barcelona. Department of Cellular Biology, Physiology and Immunology. Cerdanyola del Vallès, 08193, Spain

^3^ University of Barcelona. Department of Statistics, Barcelona, 08028, Spain

^4^ Germans Trias i Pujol University Hospital, Department of Neurosciences, Multiple Sclerosis Unit, Badalona, 08916, Spain

* emmartinez.germanstrias@gencat.cat

**Supplementary Table S1**. Enriched pathways and protein sets versus iDC in vitD3‑tolDC, rapa‑tolDC and dexa‑tolDC.

| *GSEA versus iDC* | | | | | | | |
| --- | --- | --- | --- | --- | --- | --- | --- |
| **DATABASE** | **PROTEIN SET NAME** | **Dexa-tolDC** | | | **Rapa-tolDC** | **VitD3-tolDC** | **mDC** |
| BED | Autoreactivity and multifocal inflammation | | ▲ | ▲ | | ▲ | **=** |
| GO | Nucleosome assembly | | ▲ | ▲ | | ▲ | **=** |
| BED | Induction of lymphocytes anergy | | ▲ | **=** | | ▲ | **=** |
| GO | Fibronectin binding | | ▲ | **=** | | ▲ | **=** |
| GO | Apical plasma membrane | | ▲ | **=** | | ▲ | **=** |
| GO | Nucleosome | | ▲ | **=** | | ▲ | **=** |
| GO | Positive regulation of macrophage chemotaxis | | ▲ | **=** | | ▲ | **=** |
| GO | Response to progesterone | | ▲ | **=** | | ▲ | **=** |
| GO | Response to hypoxia | | ▲ | **=** | | ▲ | **=** |
| TRRUST | REL | | ▲ | **=** | | ▲ | **=** |
| GO | Heparin binding | | ▲ | ▼ | | ▲ | **=** |
| GO | Extracellular region | | ▲ | ▼ | | ▲ | **=** |
| GO | Inflammatory response | | ▲ | ▼ | | ▲ | **=** |
| GO | Heme binding | | **=** | ▲ | | ▲ | **=** |
| GO | Nuclear nucleosome | | **=** | ▲ | | ▲ | **=** |
| TRRUST | ZFP36 | | **=** | ▲ | | ▲ | **=** |
| BED | Recruitment of cells and establishment of microenvironment | | ▲ | ▲ | | **=** | **=** |
| GO | Cell adhesive protein binding involved in bundle of His cell-Purkinje myocyte communication | | ▲ | ▲ | | **=** | **=** |
| GO | Desmosome | | ▲ | ▲ | | **=** | **=** |
| GO | Interleukin-2-mediated signaling pathway | | ▲ | ▲ | | **=** | **=** |
| TRRUST | STAT6 | | ▲ | ▲ | | **=** | **=** |
| GO | Endoplasmic reticulum membrane | | ▼ | ▼ | | **=** | **=** |
| GO | Small molecule metabolic process | | ▼ | ▼ | | **=** | **=** |
| GO | Activation of phospholipase C activity | | **=** | ▼ | | ▼ | **=** |
| GO | Drug transmembrane transport | | **=** | ▼ | | ▼ | **=** |
| KEGG | Complement and coagulation cascades | | **=** | ▼ | | ▼ | **=** |
| TRRUST | PPARG | | **=** | ▼ | | ▼ | **=** |
| GO | Endosome membrane | | ▼ | **=** | | ▼ | **=** |
| GO | T-tubule | | ▼ | **=** | | ▼ | **=** |
| GO | Protein oligomerization | | ▼ | **=** | | ▼ | **=** |
| GO | Regulation of cell communication by electrical coupling | | ▼ | **=** | | ▼ | **=** |
| GO | Negative regulation of adenylate cyclase activity | | ▼ | **=** | | ▼ | **=** |
| KEGG | Dopaminergic synapse | | ▼ | **=** | | ▼ | **=** |
| SMPDB | Malonyl-CoA decarboxylase deficiency | | ▼ | **=** | | ▼ | **=** |
| BED | Sustained inflammation | | ▲ | ▲ | | ▲ | ▼ |
| BED | Dendritic cell tolerogenicity | | ▲ | ▲ | | ▲ | ▼ |
| BED | Immunosuppressant context | | ▲ | **=** | | ▲ | ▼ |
| BED | Dendritic cell adhesion disruption | | **=** | ▲ | | ▲ | ▼ |
| GO | Cellular calcium ion homeostasis | | ▼ | ▼ | | **=** | ▼ |
| GO | Modulation by virus of host morphology or physiology | | ▼ | ▼ | | **=** | ▼ |
| GO | Regulation of insulin secretion | | ▼ | ▼ | | **=** | ▼ |
| GO | Positive regulation of cholesterol storage | | ▼ | ▼ | | **=** | ▼ |
| GO | Synaptic transmission | | ▼ | ▼ | | **=** | ▼ |
| GO | Filopodium assembly | | ▼ | ▼ | | **=** | ▼ |
| GO | Leukotriene biosynthetic process | | ▼ | ▼ | | **=** | ▼ |
| GO | Neuronal cell body | | ▼ | ▼ | | **=** | ▼ |
| GO | Plasma membrane | | ▼ | ▼ | | **=** | ▼ |
| KEGG | Gap junction | | ▼ | ▼ | | **=** | ▼ |
| PharmGKB | VEGF signaling pathway | | ▼ | ▼ | | **=** | ▼ |
| BED | Fatty acid uptake | | **=** | ▼ | | ▼ | ▼ |
| GO | Lipopolysaccharide receptor activity | | **=** | ▼ | | ▼ | ▼ |
| GO | Toll-like receptor 2 signaling pathway | | **=** | ▼ | | ▼ | ▼ |
| GO | Innate immune response | | **=** | ▼ | | ▼ | ▼ |
| GO | Toll-like receptor 4 signaling pathway | | **=** | ▼ | | ▼ | ▼ |
| GO | Cholesterol transport | | **=** | ▼ | | ▼ | ▼ |
| GO | Positive regulation of lipopolysaccharide-mediated signaling pathway | | **=** | ▼ | | ▼ | ▼ |
| GO | Extrinsic apoptotic signaling pathway | | **=** | ▼ | | ▼ | ▼ |
| GO | Triglyceride catabolic process | | **=** | ▼ | | ▼ | ▼ |
| GO | Positive regulation of tumor necrosis factor production | | **=** | ▼ | | ▼ | ▼ |
| GO | Cellular response to lipoteichoic acid | | **=** | ▼ | | ▼ | ▼ |
| GO | Activation of cysteine-type endopeptidase activity involved in apoptotic signaling pathway | | **=** | ▼ | | ▼ | ▼ |
| GO | Toll-like receptor signaling pathway | | **=** | ▼ | | ▼ | ▼ |
| GO | Regulation of cytokine secretion | | **=** | ▼ | | ▼ | ▼ |
| GO | Integral component of plasma membrane | | **=** | ▼ | | ▼ | ▼ |
| GO | Lipid particle | | **=** | ▼ | | ▼ | ▼ |
| GO | Lipopolysaccharide receptor complex | | **=** | ▼ | | ▼ | ▼ |
| KEGG | PPAR signaling pathway | | **=** | ▼ | | ▼ | ▼ |
| KEGG | Endocrine and other factor-regulated calcium reabsorption | | **=** | ▼ | | ▼ | ▼ |
| TRRUST | ETS1 | | **=** | ▼ | | ▼ | ▼ |
| GO | Lipopeptide binding | | ▼ | **=** | | ▼ | ▼ |
| GO | Beta-2-microglobulin binding | | ▼ | **=** | | ▼ | ▼ |
| GO | Exogenous lipid antigen binding | | ▼ | **=** | | ▼ | ▼ |
| GO | Endogenous lipid antigen binding | | ▼ | **=** | | ▼ | ▼ |
| GO | Antigen processing and presentation, exogenous lipid antigen via MHC class Ib | | ▼ | **=** | | ▼ | ▼ |
| KEGG | Thyroid hormone synthesis | | ▼ | **=** | | ▼ | ▼ |

Green arrow: upregulation of said set; Yellow bar: unchanged regulation of said set; Red arrow: downregulation of said set. BED: Biological Effectors Database; GO: Gene Ontology; KEGG: Kyoto Encyclopedia of Genes and Genomes; PharmGKB: Pharmacogenomics Knowledgebase; SMPDB: Small Molecule Pathway Database; TRRUST: Transcriptional Regulatory Relationships Unraveled by Sentence-based Text-mining.
